# Health economic evaluations of fecal microbiota transplantation for non-clostridioides difficile related diseases: a systematic review

**DOI:** 10.1186/s13561-025-00698-5

**Published:** 2025-12-16

**Authors:** Qiran Wei, Yingcheng Wang, Mingjun Rui, Joyce H.S. You

**Affiliations:** School of Pharmacy, Faculty of Medicine, The Chinese University of Hong Kong, 8/F, Lo Kwee-Seong Integrated Biomedical Sciences Building Shatin, N.T., Shatin, Hong Kong SAR China

**Keywords:** Fecal microbiota transplantation, Health economic evaluation, Systematic review, Inflammatory bowel disease, Fecal donor screening, Urinary tract infection

## Abstract

**Objectives:**

This study aimed to conduct a systematic review on health economic evaluations of fecal microbiota transplantation (FMT) for non-Clostridioides difficile infection (non-CDI) related diseases.

**Methods:**

A systematic search of literature was conducted up to October 2025 in MEDLINE (Ovid), Embase (Ovid), APA PsycINFO, Web of Science, Scopus, and CINAHL Ultimate. Studies inclusion criteria were: (1) Non-CDI related diseases; (2) FMT as a treatment; (3) health economic evaluations; and (4) original research articles published in English. The quality of included studies was evaluated using the Consolidated Health Economic Evaluation Reporting Standards (CHEERS) checklist.

**Results:**

Six studies were included. All studies were conducted from the healthcare provider’s perspective, and one additionally considered the societal perspective. Three studies (50%) were cost analyses (*n* = 1 inflammatory bowel disease (IBD); *n* = 2 fecal donor screening for treatment of melanoma and metabolic syndrome-associated diseases), two (33%) were cost-effectiveness analyses (IBD), and one (17%) was cost-consequence analyses (urinary tract infection (UTI) caused by multidrug-resistant organisms (MDROs)). The studies were classified as excellent (*n* = 1), very good (*n* = 2), and insufficient (*n* = 3). Four studies (67%) reported that FMT was a potentially cost-saving intervention for IBD (*n* = 3) and UTI (*n* = 1). One cost-effectiveness study of 3 FMT regimens for IBD treatment showed the number FMT administrations was influential to total treatment cost.

**Conclusions:**

The included health economic evaluations of FMT for non-CDI related diseases are scarce and quality is diverse. The small number of analyses highlights the research gap of health economic evaluation of FMT for non-CDI related diseases with positive clinical benefits.

**Supplementary Information:**

The online version contains supplementary material available at 10.1186/s13561-025-00698-5.

## Introduction

The human intestine is inhabited by a vast array of microorganisms, and these microbes play a crucial role in maintaining overall human health [1, 2]. Dysbiosis, a disturbance of the gut microbiota population, is associated with the pathogenesis and progression of numerous diseases [3]. While gut dysbiosis is most commonly reported in gastrointestinal disorders, such as Clostridioides difficile infection (CDI) and inflammatory bowel disease (IBD) [4], increasing evidence have linked gut microbiota imbalances to diseases affecting the liver [5] and brain [6].

Fecal microbiota transplantation (FMT) has emerged as a promising therapeutic intervention to restore the gut microbiome. This procedure involves the transfer of stool, along with the associated microbial communities, from a healthy donor to a recipient’s gastrointestinal tract [7]. FMT has been widely adopted in clinical practice for the treatment of recurrent CDI, and it has shown significant improvement in clinical cure [8–10]. FMT has also been recommended for severe and fulminant CDI cases which are refractory to antimicrobial treatment, especially when surgical options are not viable [8]. Systematic reviews have reported the cost-effectiveness of FMT for CDI treatment, supporting its application in routine clinical care [11, 12].

FMT also showed potentials in treatment of some non-CDI related diseases (such as IBD, irritable bowel syndrome, obesity and metabolic syndrome, and non-alcoholic fatty liver disease (NAFLD)) [13–18], although the use of FMT for these indications remains confined to research settings [19]. These non-CDI related diseases imposed a substantial global health and economic burden. IBD affected over 6.8 million people worldwide [20], and the direct medical costs for IBD patients were more than three times higher than for those without IBD (USD 22,987 vs. USD 6,956 per patient-year) in the US [21]. The global prevalence of NAFLD was 25% [22], and patients with NAFLD were at higher risk of all-cause mortality versus individuals without NAFLD (HR = 1.34; 95% CI = 1.17–1.54) [23]. Studies of FMT have reported promising improvement in treatment outcomes of some non-CDI related diseases. In patients with IBD, FMT plus standard treatment was associated with significantly higher clinical remission rates than standard treatment alone (RR = 1.70; 95% CI = 1.12–2.56) [24]. In patients with NAFLD, the mean fat attenuation values significantly decreased from 278.3 dB/m to 263.9 dB/m in patients receiving FMT plus standard treatment (*p* = 0.049) [13]. The impact of FMT-related clinical benefits on health economics were also emerging. Health economic evaluations have reported FMT to be cost-saving or cost-effective for IBD treatment [25, 26]. Nevertheless, FMT incurs significant costs (including expenses for donor screening, laboratory preparation, and administration [27]) and such costs impose uncertainty in the potential cost-effectiveness of FMT. Health economic evaluation is a common tool to assess the values of clinical and economic outcomes associated with healthcare interventions. Health economic evaluations of FMT provided evidence to assist clinical researchers, healthcare providers and administrators in the process of informed decision-making on resource allocation for clinical research and implementation of FMT treatment. There is no published systematic review on existing health economic literature of FMT for non-CDI diseases. This study aims is to conduct a systematic review on health economic evaluations of FMT for non-CDI related diseases and evaluate the quality of included studies. Our findings are anticipated to provide a comprehensive summary of current evidence, identify research gaps, and inform future health economic evaluations regarding the use of FMT beyond CDI.

## Methods

### Search strategy

A systematic literature search was conducted in the MEDLINE (Ovid), Embase (Ovid), APA PsycINFO, Web of Science, Scopus, and CINAHL Ultimate, up to October 2025. We conducted a preliminary search to identify potentially relevant search terms for FMT, such as “fecal microbiota transplant”, “donor feces infusion”, and “intestinal microbiota transfer”. The search terms for FMT were then combined with search terms for “health economic evaluation”. References of the included studies were also searched manually to identify any additional relevant studies. The complete set of electronic database search strategy and the Preferred Reporting Items for Systematic Reviews and Meta-Analyses (PRISMA) 2020 checklist are summarized in the Supplementary Materials 1. The protocol of this systematic review was registered at PROSPERO (registration number: CRD42024598362).

### Selection criteria

Publications were eligible if the studies met the following inclusion criteria: (1) non-CDI related disease was examined; (2) FMT was evaluated as a treatment; (3) health economics analysis was conducted, including cost, cost-effectiveness, and cost-consequence analyses; and (4) the report was published as an original research article in English. We excluded articles if the study: (1) focused on CDI; (2) did not evaluate FMT as a treatment; (3) did not conduct health economics analysis; or (4) was published in the form of review, case report, protocol, editorial, comments, letter, conference abstract, or poster presentation.

### Study selection process

Studies identified as potentially eligible for inclusion were imported into EndNote. After duplicates were removed, three reviewers (QW, YW and MR) independently conducted two rounds of literature screening to determine whether each study met the eligibility criteria. In the first phase, title and abstract were evaluated for preliminary screening and irrelevant studies were excluded. In the second round, full-text review was performed to determine the study’s eligibility according to the inclusion and exclusion criteria. In each round, discrepancies among reviewers were solved through discussion or by consulting the fourth reviewer (JHSY). The entire selection process was conducted following the PRISMA statement for systematic review [28, 29].

### Data extraction

Three reviewers (QW, YW and MR) worked independently to extract the following information: (1) general characteristics: title, first author, publication year, and country; (2) patient characteristics: disease, intervention and comparator; (3) study methodology: type of health economic evaluation, study design (model-based or trial-based), study perspective, time horizon, primary outcomes, willingness-to-pay (WTP) and sensitivity analysis methods; (4) model characteristics (applicable for model-based studies); (5) trial characteristics (applicable for trial-based studies); (6) health economics results: Incremental costs, health outcome (clinical event or quality-adjusted life-year (QALY)) gains, incremental cost-effectiveness ratios (ICERs), influential parameters identified by sensitivity analyses, and probabilistic sensitivity analysis results. Disagreements in data extraction were discussed to reach a consensus.

### Quality assessment

The quality assessment of all included studies was conducted using the Consolidated Health Economic Evaluation Reporting Standards (CHEERS) 2022 checklist [30]. This checklist includes 28 items across seven sections: title, abstract, introduction, methods, results, discussion, and other relevant information (source of funding and conflict of interest). The included studies were evaluated for each item of the CHEERS checklist, categorized as “fully reported”, “partially reported”, or “not reported”. The grading system used in the present assessment was adopted from prior systematic reviews [31, 32]: 1 point was assigned to the items that were fully reported, 0.5 points for partially reported, and 0 points for not reported. The total score was converted into percentages, and each included study was categorized into one of four categories: “Excellent” (≥ 85%), “very good” (70%−84%), “good” (55%−69%), and “insufficient” (< 55%). The assessment was conducted independently by three reviewers (QW, YW and MR), and any discrepancies were resolved by discussion and consensus with the fourth reviewer (JHSY).

### Data analysis and presentation

The screening and selection process, including the number of studies identified and excluded, were shown in a flowchart. The descriptive characteristics, and findings of cost analysis, cost-effectiveness analysis, and cost-consequence analysis, as well as quality assessment of the included studies were summarized. The FMT strategy was considered cost-effective if (1) it was more effective and less costly than the comparator, or (2) it was more effective at a higher cost with an ICER below the WTP threshold.

## Results

### Study selection results

**Fig. 1:**
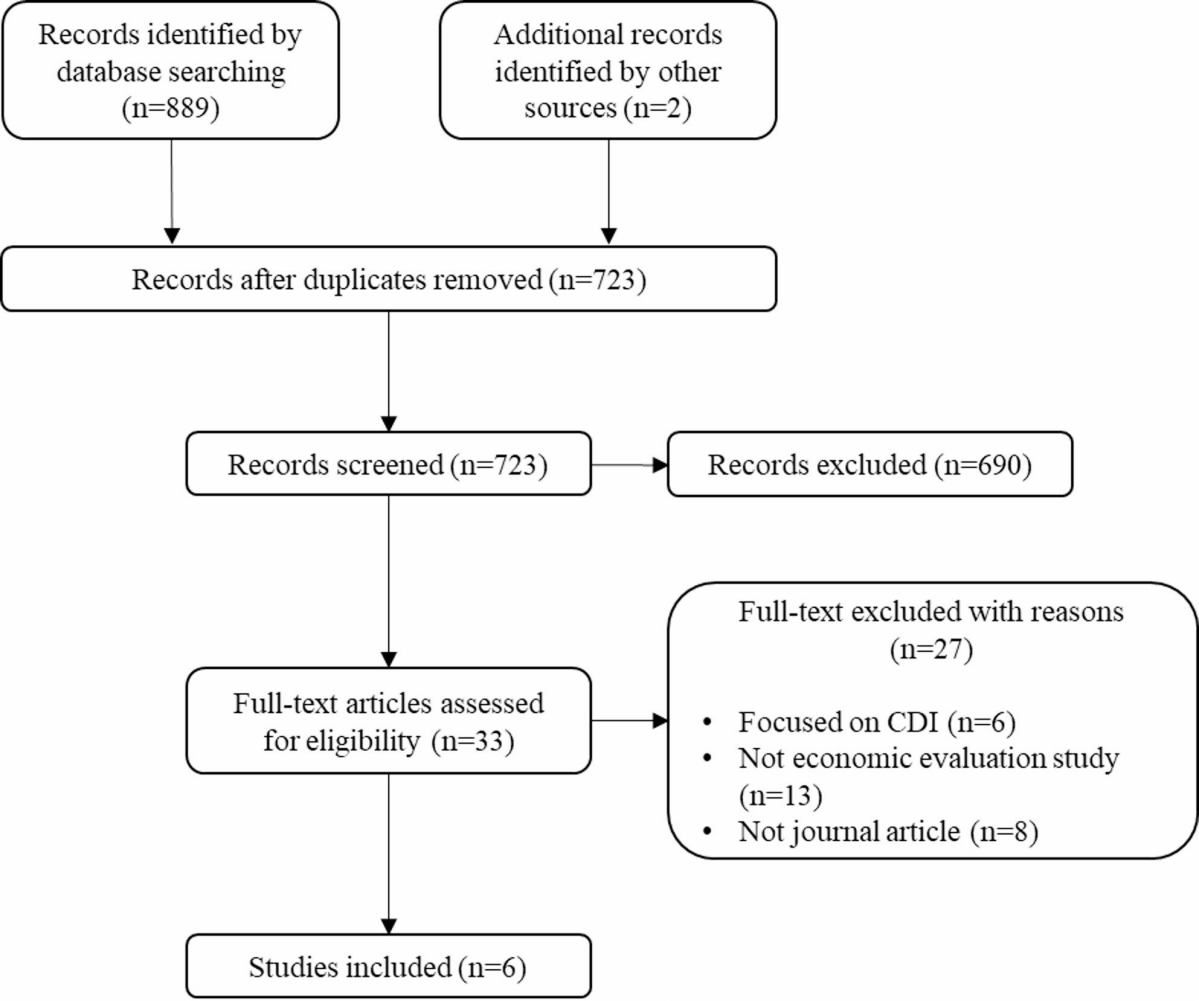
PRISMA flow diagram for study inclusion

The systematic search identified 889 records in target databases and 2 records by manual searches. After removal of duplicates, 723 records were screened by the titles and abstracts, identifying 33 papers for full-text review. A total of 27 studies were excluded on the full-text review: 6 focused on CDI, 13 did not conduct health economics analysis, and 8 were not journal article. Six studies met the selection criteria and were included in this review [25, 26, 33–36]. The PRISMA flow diagram of the search and selection processes is presented in Fig. 1.

### Study characteristics

The characteristics of 6 included studies are summarized in Table 1. All evaluations were published after 2017. Three studies were conducted in the United States [25, 36], two in China[26, 33], one in Canada [35], and one in Denmark [34]. Three studies (50%) targeted at IBD [25, 26, 33], and one (17%) on PD-1 refractory melanoma [36], one (17%) on urinary tract infections (UTIs) caused by multidrug-resistant organisms (MDROs) [34] and one (17%) on metabolic syndrome-associated diseases [35]. The interventions evaluated were FMT plus conventional treatment and FMT monotherapy, comparing with conventional treatment.

**Table 1:** Study characteristics of 6 included studies

Authors (year); country	Intervention and comparator	Type of health economic evaluation; perspective	Study design; timeframe	Primary outcomes	Willingness to pay	Base-case and sensitivity analyses	Quality category
Inflammatory bowel disease							
Zhang et al. (2024) [33]; China	Interventions: (1) WMT^a^ combined with mesalazine, (2) WMT Comparator: Infliximab	Cost analysis; health care provider’s perspective	Trial-based study (*n* = 60); 1 year	Cost	Not reported	Base-case analysis: WMT + mesalazine: CNY 29,756 (USD 4,146) per year, WMT monotherapy: CNY 19,598 (USD 2,730) per year, Infliximab: CNY 46,414 (USD 6,466) per year Sensitivity analysis: not reported	Insufficient
Yao et al. (2023) [25]; United States	Interventions: FMT^b^ (with 3, 6, and 41 administrations) plus standard treatment Comparator: Standard treatment (mesalazine)	Cost-effectiveness analysis; health care provider’s perspective	Model-based study; 10 years	Cost, QALY^c^ ICER^d^	50,000 USD/QALY	Base-case analysis: FMT more effective than standard treatment (by 0.068 QALYs) 3 FMT administrations: less costly than standard treatment (by USD − 3,868) 6 FMT administrations: more costly than standard treatment (by USD 670), ICER = 9,918 USD/QALY 41 FMT administrations: more costly than standard treatment (by USD 60,542), ICER = 895,786 USD/QALY One-way sensitivity analysis: Most influential factor: Relative risk of achieving remission with FMT therapy (thresholds: >1.711 and > 1.977 for 3 and 6 FMT administrations to be cost-effective, respectively) Probabilistic sensitivity analysis: Probabilities of standard treatment plus FMT therapy to be cost-effectiveness were 91%, 67%, and 0% with 3, 6 and 41 administrations, respectively.	Excellent
Zhang et al. (2017) [26]; China	Intervention: FMT plus conventional therapy Comparator: Conventional therapy (including 5-aminosalicylic acid, probiotics, corticosteroids, antibiotics, immunomodulators, biologics, traditional Chinese medicine, nutrition and surgery)	Cost-effectiveness analysis; health care provider’s and societal perspective	Trial-based study (*n* = 104); 1 year	Cost, QALY, ICER	141,240 CNY/QALY	Base-case analysis: Healthcare perspective: FMT plus conventional therapy less costly (by CNY − 25,814 (USD − 3,596)) and more effective (by 0.139 QALYs) than conventional therapy alone, ICER = −185,712 CNY/QALY (−25,873 USD/QALY) Societal perspective: FMT plus conventional therapy less costly (by CNY − 28,831 (USD − 4,017)) and more effective (by 0.139 QALYs) than conventional therapy alone, ICER = −207,417 CNY/QALY (−28,897 USD/QALY) Probabilistic sensitivity analysis: Probabilities of FMT plus conventional therapy to be cost-effectiveness were 73% (healthcare perspective) and 75% (societal perspective).	Very good
PD-1 refractory melanoma							
Fortman et al. (2023) [36]; United States	Intervention: Fecal donors screening Comparator: None	Cost analysis; health care provider’s perspective	Trial-based study (*n* = 29); time frame not reported	Cost	Not reported	Base-case analysis: Cost of screening per donor/recipient: USD 2,260 (pre-COVID) and USD 2,460 (post-COVID). Sensitivity analysis: not reported	Insufficient
Urinary tract infections caused by multidrug-resistant organisms							
Baek et al. (2023) [34]; Denmark	Intervention: FMT plus conventional therapy Comparator: Pre-FMT conventional therapy (antibiotics)	Cost-consequence analysis; health care provider’s perspective	Trial-based study (*n* = 5); Median 126 days (range 60–320 days)	Cost, number of UTI episode, hospitalization days, health contacts (admission, outpatient visit, telephone contact emergency room visit)	Not reported	Base-case analysis (outcome changes from pre-FMT to post-FMT): Median UTI episodes per patient: Decreased from 4 to 0 Median length of hospitalization: Decreased from 15 days to 4 days Median hospital contacts per patient: Decreased from 28 to 11 Average monthly cost per patient: Decreased from USD 6,687 to USD 1,574 (FMT cost not included) Sensitivity analysis: The FMT strategy remained to be cost-saving when excluding the patient with the lowest costs or the one with the highest costs.	Very good
Metabolic syndrome-associated diseases							
Craven et al. (2017) [35]; Canada	Intervention: Fecal donors screening Comparator: None	Cost analysis; health care provider’s perspective	Trial-based study (*n* = 46); not reported	Cost	Not reported	Base-case analysis: Cost for screening one donor: USD 439 Sensitivity analysis: not report	Insufficient

^a^WMT: washed microbiota transplantation

^b^FMT: fecal microbiota transplantation

^c^QALY: quality-adjusted life year

^d^ICER: incremental cost-effectiveness ratio

Two included studies were cost-effectiveness analyses with ICER as the main outcome [25, 26], and one was a cost-consequence analysis [34] where cost and improved effectiveness were the primary outcomes. The remaining three (50%) studies were cost analyses, evaluating the costs of IBD treatment [33] and fecal donors screening [35, 36]. In terms of study design, five (83%) reports were trial-based studies [26, 33–36] and one (17%) was model-based study [25]. All studies were conducted from the perspective of the health care provider, and one study also included a societal perspective [26]. Four (67%) studies reported the study time horizon, ranging from 60 days to 10 years [25, 26, 33, 34]. Three (50%) studies performed sensitivity analyses [25, 26, 34], including one-way sensitivity analysis and probabilistic sensitivity analysis.

### Study quality

The quality assessment findings based upon the CHEERS 2022 checklist are presented in Supplementary Materials 2. One (17%) included study [25] was categorized as excellent, two (33%) [26, 34] as very good, three (50%) as insufficient [33, 35, 36]. The mean CHEERS score was 17.6 (SD 4.89, range 12.5–25.0). Of the 28 CHEERS items, all studies (100%) have fully reported 9 items: (1) Setting and location, (2) comparators, (3) selection of outcomes, (4) rationale and description of model, (5) analytics and assumptions of model, (6) summary of main results, (7) study findings, limitations, generalizability, and current knowledge, (8) source of funding, and (9) conflicts of interest. There were 14 items fully reported in ≥ 50% of the studies: (1) Title, (2) abstract, (3) background and objectives, (4) health economic analysis plan, (5) study population, (6) perspective, (7) measurement of outcomes, (8) valuation of outcomes, (9) measurement and valuation of resources and costs, (10) time horizon, (11) characterizing heterogeneity, (12) characterizing uncertainty, (13) study parameters, and (14) effect of uncertainty. The remaining 5 items were fully reported in < 50% of the studies: (1) Discount rate, (2) currency, price date, and conversion, (3) characterizing distributional, (4) approach to engagement with patients and others affected by the study, and (5) effect of engagement with patients and others affected by the study.

### FMT for Non-CDI related diseases

#### FMT for IBD

IBD, including ulcerative colitis and Crohn’s disease, is a life-long disease characterized by chronic mucosal inflammation of the gastrointestinal tract. The efficacy and safety of FMT for treatment of IBD have been showed in clinical trials that FMT is associated with improved clinical remission [16, 18].

A trial-based study reported the cost-effectiveness analysis of FMT by comparing the outcomes before and after applying FMT for 104 patients with moderate to severe active IBD (33 ulcerative colitis; 71 Crohn’s disease) [26]. Both medical costs (hospitalization and outpatient expenses) and non-medical costs (lost work time for patients and their family members) were analyzed from the healthcare and societal perspectives in the timeframe of 1-year pre-FMT and 1-year post-FMT. Health utility was assessed by EuroQol-5 at baseline pre-FMT and 1-year post-FMT. The findings showed cost-savings (CNY 25,814 (USD 3,596) from medical perspective; CNY 28,831 (USD 4,017) from societal perspective) and higher QALYs gained (by 0.139 QALYs) in the FMT group versus standard therapy (including 5-aminosalicylic acid, probiotics, corticosteroids, antibiotics, immunomodulators, biologics, traditional Chinese medicine, nutrition, and surgery). Probabilities of FMT to be cost-effectiveness were 73% (healthcare perspective) and 75% (societal perspective) at the WTP threshold of 141,240 CNY/QALY. The study also reported net monetary benefit of different subgroups (by age, gender, smoking status, and disease subtype). The results suggested that younger patients (under 24 years), females, non-smokers, and patients with Crohn’s disease were likely to gain greater net benefits from FMT.

A cost-effectiveness analysis examined the outcomes of standard treatment (mesalazine) with and without FMT therapy for patients with mild-to-moderate active ulcerative colitis using a 10-year Markov model [25]. The hypothetical patients initially received an 8-week induction treatment with mesalazine, with or without FMT therapy. If patients remained in a non-remission state, treatment was progressively escalated to include prednisolone, methylprednisolone, azathioprine, infliximab, adalimumab, vedolizumab, and surgery. Standard treatment with FMT gained higher QALY than standard treatment alone (by 0.068 QALYs). Costs were considered from the perspective of the US healthcare provider. Three cost levels for FMT therapy were estimated for regimens with 3, 6 and 41 administrations. When FMT was administered 3 times per course, the total cost of FMT plus standard treatment was cost-saving when compared to standard treatment alone (by USD 3,868). If FMT was 6-time and 41-time per course, the incremental cost (versus standard treatment) was USD 670 and USD 60,542 respectively. Using a WTP threshold of 50,000 USD/QALY, the 3-time and 6-time courses FMT with standard treatment were accepted as cost-effective, and the 41-time course was not cost-effective. The one-way sensitivity analysis identified the relative risk of achieving remission with FMT as the most significant factor influencing the ICERs. The probabilistic sensitivity analysis indicated that the probabilities to be cost-effective were 91% and 67% for FMT 3-time and 6-time courses, respectively.

A cost analysis investigated the direct medical costs of three IBD treatment strategies from the perspective of the health care provider in China: (1) Infliximab, (2) washed microbiota transplantation (WMT) monotherapy, and (3) WMT with mesalazine [33]. WMT is a method to prepare FMT by the automatic washing process to certain bacterial fragments, pro-inflammatory metabolites, soluble molecules and virus [37]. The direct medical costs for WMT monotherapy and WMT with mesalazine were collected retrospectively from a cohort of 60 IBD patients (36 ulcerative colitis; 24 Crohn’s disease) who had achieved sustainment for at least one year by the WMT regimens (26 patients on WMT monotherapy; 34 patients on WMT plus mesalazine). The cost per year of WMT with mesalazine was higher than that of WMT monotherapy (CNY 29,756 (USD 4,146) versus CNY 19,598 (USD 2,730) per year; *p* = 0.001). The direct medical cost of infliximab standard therapy was estimated based upon the infliximab treatment protocol including costs of infliximab, monitoring tests and hospitalization. The estimated cost per year of infliximab standard therapy was CNY 46,414 (USD 6,466).

#### FMT for PD-1 refractory melanoma

Immune-check-point inhibitors is the immunotherapy targeting cytotoxic T-lymphocytes associated antigen 4 and programmed death 1 (PD-1) inhibitory immune checkpoints. Studies have showed that gut microbiome is associated with response to anti-PD-1 immunotherapy in melanoma patients [38, 39]. The administration of responder-derived FMT was found to re-sensitize patients with primary refractory melanoma to pembrolizuma (anti-PD-1 immunotherapy) [40, 41]. The cost of donor screening contributed to the major part of FMT cost [42]. A cost analysis reported the screening costs associated with selecting FMT donors for the treatment of PD-1 refractory melanoma patients in the US [36]. Both PD-1 responders in sustained remission (as donors) and patients with PD-1 refractory (as recipients) underwent medical evaluations and screenings. Extra infectious serological tests in melanoma patients (including cytomegalovirus, Epstein Barr virus, herpes simplex virus type 1, herpes simplex virus type 2, human herpesvirus 6, human polyomavirus 2) led to higher screening cost than in healthy donors from the public stool banks [36, 43]. Following the onset of COVID-19, COVID-19 diagnostic assays were added to test stool and nasopharyngeal specimens. Patients with suspected COVID-19 infection were excluded during the post-COVID phase. Of the 29 PD-1 responders screened, 14 were eligible to regularly donate for the program, resulting in an acceptance rate of 48%. The cost of screening per donor/recipient was USD 2,260 (pre-COVID) and USD 2,460 (post-COVID).

#### FMT for utis caused by Multidrug-resistant organisms

Case series suggested association of FMT with potential clinical improvement in patients with recurrent UTIs caused by MDROs [44–47]. A cohort trial-based early health economic evaluation on cost and consequences of five Danish consecutive patients with MDRO-related UTIs who were treated with FMT plus conventional therapy (antibiotics) [34]. Clinical health outcomes were derived from electronic patient records, including number of UTI episodes, hospitalization days, and health contacts (admission, outpatient visit, telephone contact emergency room visit). Inpatient and outpatient costs were estimated by Danish diagnosis-related group tariffs, and the cost of FMT was not included. The median follow-up was 126 days (range 60–320 days). Pre-FMT data collection period was extended to the same length of follow-up post-FMT. Compared to the outcomes of pre-FMT, the post-FMT median number of UTI episodes per patient dropped from 4 to 0, the median hospitalization days decreased from 15 days to 4 days, the median number of hospital contacts per patient decreased from 28 to 11, and the average monthly cost per patient decreased from USD 6,687 to USD 1,574. The FMT strategy remained to be cost-saving when excluding the patient with the lowest costs or the one with the highest costs.

#### FMT for metabolic Syndrome-associated diseases

FMT was associated with alleviating the features of metabolic syndrome, including improvement in hemoglobin A1c and high-density lipoprotein cholesterol [48, 49]. A cost analysis on screening costs of selecting FMT donors for the treatment of metabolic syndrome-related diseases was evaluated in a trial-based study in Canada [35]. This cost analysis provided a minimum estimate of FMT costs for clinical institution. Exclusion criteria for FMT donor screening in metabolic syndrome-related diseases were stricter than those applied in the public stool bank, including body mass index more than 25, abnormal metabolic parameters, recent antibiotic use, family history of diabetes or coronary disease, and any known transmissible agent. A total of 46 potential donors underwent the medical history reviews and physical examinations, of which 23 were cleared for further blood, urine, stool, and throat/rectal swab screenings. Five donors were eligible per testing results, resulting in an acceptance rate of 11%. Of these five individuals, four were excluded subsequently for acute gastroenteritis and travel to tropical countries, and only one volunteer regularly donated for the program. The total cost for the full screening process was USD 439 per person, and the cost for those excluded after an initial history and physician examination was USD 150 per person.

## Discussion

This is the first comprehensive systematic review on the published health economic evaluations of FMT for non-CDI related diseases. The publication date of the included studies spread between 2017 and 2024, with 2 published before 2021 and 4 published after 2021, indicating the increasing health economic research interest of FMT for medical conditions other than CDI. The target diseases included IBD, melanoma, UTIs and metabolic syndrome-associated diseases. Most studies were conducted in high-income countries (the United States, Denmark, and Canada) [25, 34–36], and the remaining two studies were carried out in upper-middle-income countries (China) [26, 33]. The quality of health economic evaluations is a critical factor for the reliability of result estimates, comparability across studies and the generalizability of findings. Quality of the six included studies was diverse. Three studies were ranked as very good to excellent [25, 26, 34] according to the CHEERS checklist, and these studies (including cost-effectiveness and cost-consequence analyses) reported that FMT was cost-saving (and effective) or cost-effective for non-CDI related diseases (IBD and MDRO-related UTI). The remaining three studies [33, 35, 36] (ranked as insufficient) were cost analyses, and did not provide comprehensive cost-effectiveness findings to inform decision-making. Common inadequately reporting CHEERS items (reported in < 50% of the studies) were discount rates; currency, price date, and conversion; distributional effects; approach to engagement with patients and others affected by the study; and effect of engagement with patients and others affected by the study.

Two included studies were cost analyses focusing on the costs associated with fecal donor screening, and the cost per donor screening ranged between USD 439 and USD 2,460 [35, 36]. The cost drivers of screening cost were the screening items, cost per laboratory test and screening success rate. Some tests were priced at a lower cost (or even zero cost for tests conducted at provincial public health laboratories in Canada) in the metabolic syndrome-associated diseases study [35] than those of the US study of PD-1 refractory melanoma [36]. The success rates for identifying eligible donors were modest in these two cost analyses. The overall screening efficiency was 48% (14/29) in the PD-1 refractory melanoma study, and it was challenged by the donor inclusion criteria of PD-1 responders in sustained remission and extensive list of infectious serological tests on exclusion criteria [36]. In the metabolic syndrome-associated diseases study [35], the low successful screening rate (11%; 5/46) was mainly attributable to the highly metabolic function-specific inclusion criteria [35]. FMT donor recruitment success rate was highly specific to the target disease and corresponding selection criteria, and the costs of laboratory testing also varied across countries.

Four included studies reported that FMT was a potentially cost-saving intervention for treating non-CDI related medical conditions (IBD [25, 26, 33] and MDRO-related UTI [34]). One of these studies used Markov modeling to simulate outcomes from the US payer’s perspective and found that the use of FMT lead to higher QALYs for IBD treatment. The study further estimated cost-effectiveness of 3 FMT regimens for IBD treatment, and found the total treatment cost was highly sensitive to the number FMT administrations [25]. Two trial-based health economic studies of FMT for IBD were conducted by the same research team [26, 33]. FMT (with standard treatment) was reported to gain higher QALYs at lower cost in 104 IBD patients. The cost savings were driven by fewer admissions and reduction of medical treatments (particularly corticosteroids and biologics) [26]. The same research team further examined the efficacy and safety of WMT as a recently developed preparation of FMT [37] and further examined the cost of WMT for IBD in a trial-based study of 60 IBD patients [33]. WMT was reported to generate cost savings versus standard treatment. The standard infliximab regimen used in the study represented a conservative estimate of treatment costs. Patients who did not respond to infliximab would increase the dose, shorten dosing intervals, or switch to alternative therapies in real-world practice, all of which further increased treatment costs of non-responsive IBD cases [33]. A trial-based cost-consequence analysis of 5 patients with MDRO-related UTI showed significantly reductions in recurrent infections, hospitalization days, health contacts, and monthly costs following FMT [34]. Despite the small sample size, the study demonstrated statistically significant decreases in hospital admissions and associated costs, indicating considerable economic benefits generated by the clinical impact of FMT. It is important to note that the study on MDRO-related UTI did not include the FMT cost and only focused on hospital-related costs. When the cost of FMT was higher than USD 5,113, FMT was no longer a cost-saving strategy.

This review identified several research gaps in the health economic evaluations of FMT for non-CDI related diseases. Firstly, few types of targeted non-CDI related diseases were examined in the included studies. Previous clinical trials have reported that FMT is a promising therapeutic option in IBD [16, 18], irritable bowel syndrome [14], obesity and metabolic syndrome [15, 17], and non-alcoholic fatty liver disease [13]. These positive clinical findings on FMT suggest the needs for further health economic evaluations on the potential cost-effectiveness of FMT versus the disease-specific standard treatments for a broader array of non-CDI related conditions. Secondly, the included studies focused on adult patients, and there is a lack of health economic evaluations of FMT for non-CDI related diseases in the pediatric populations. A recent study found that using FMT as first-line therapy for children with active Crohn’s disease has showed improvement in induction and maintenance of remission, as well as mucosal healing [50]. A clinical trial (ClinicalTrials.gov, registered number NCT05679622) is currently underway to evaluate the use of FMT for refractory pediatric ulcerative colitis. The findings from these trials provide new insights into the use of FMT in managing pediatric IBD patients, and health economic evaluations of FMT for pediatric IBD treatment are warranted. Thirdly, there is a lack of health economic evaluations conducted in middle- and low-income countries. Healthcare systems in these countries often face resource limitations, and the cost-effectiveness of FMT is likely to be different from those in high-income settings due to differences in healthcare infrastructure, accessibility, and affordability. The health economic findings of FMT in these contexts provide evidence for optimizing resource allocation and improving access to cost-effective therapies. Lastly, different FMT delivery routes, dosages, frequencies, donor numbers, and stool types influence both costs and effectiveness. A meta-analysis of irritable bowel syndrome found that FMT via nasojejunal tube, colonoscopy, and gastroscopy showed significant improvements in overall symptoms, whilst FMT via oral capsules caused more adverse reactions [51].

Despite the limited number of included studies and the heterogeneity of diseases managed by FMT, this systematic review provides important evidence on the potential health economic implications of FMT for diseases other than CDI. The FMT for metabolic syndrome-associated diseases and PD-1 refractory melanoma required disease-specific donor selection criteria, and therefore increased donor screening costs and lower success rates for identifying eligible donors [35, 36]. For IBD and MDRO-related UTI, the health economic findings showed FMT to improve QALY gained, reduce hospitalizations and healthcare resource utilization, and achieve cost-effectiveness or cost savings [25, 26, 33, 34]. For the quality of health economic evaluation, only three of the six included studies were ranked good or above. As indicated by the quality assessment findings, future economic evaluations of FMT on non-CDI indications should strengthen methodological rigor and transparency, particularly by clearly reporting discount rate, currency and price date conversions, distributional effects, and engagement with patients and others. Clinical studies of FMT have reported promising improvements in treatment outcomes for various non-CDI related diseases (IBD, melanoma, MDRO-related UTI, irritable bowel syndrome, obesity and metabolic syndrome, non-alcoholic fatty liver disease). Our findings highlight the gaps in current evidence that the health economic evaluations of FMT for non-CDI related disease are scarce in quantity and quality. The cost-effective use of FMT for non-CDI indications remains in its infancy state. To adequately address research gaps identified above, future research should prioritize large-scale randomized controlled trials that rigorously evaluate the clinical efficacy of specific FMT regimens (including delivery routes, dosages, frequencies, donor numbers, and stool types) for each non-CDI indication to generate high-level clinical evidence. Further economic evaluations based upon the high-level clinical evidence are warranted to provide cost-effectiveness information for clinicians, patients and decision-makers for informed decisions about incorporation of FMT into the clinical treatment practice of non-CDI indications.

We conducted a comprehensive database search and manual search, following the PRISMA guidelines. However, there were several limitations in our study. The search strategy included only studies published in English, which may have led to the exclusion of relevant studies in other languages. Also, five of the six included studies [26, 33–36] were performed in a single center, which limit the generalizability of the findings to other settings or populations. The systematic review might be limited by publication bias, as studies with positive cost-effective results are more likely to be published.

## Conclusion

Despite growing clinical evidence supporting the clinical benefits of FMT for non-CDI related diseases, published health economic evaluations in this area remain scarce and the quality was diverse. The small number of included analyses highlights the research gap for further health economic evaluations of FMT for non-CDI related diseases with positive clinical benefits.

## Supplementary Information

Supplementary Material 1.

Supplementary Material 2.

## Data Availability

No datasets were generated or analysed during the current study.
