# Supplementary material for: Health economic evaluations of fecal microbiota transplantation for non-clostridioides difficile related diseases: a systematic review
Source: Health Econ Rev. 2025 Dec 16;15:103. doi: 10.1186/s13561-025-00698-5 (PMC12709682; doi:10.1186/s13561-025-00698-5)
Supplement: Supplementary file 2 — Supplementary Material 2. [file 13561_2025_698_MOESM2_ESM.docx]

**Supplementary Materials 2: CHEERS Checklist Assessment**

| Items | Zhang et al (2024) [29] | Fortman et al (2023) [32] | Baek et al (2023) [21] | Yao et al (2022) [22] | Craven et al (2017) [31] | Zhang et al (2017) [30] | Studies fulfilled the item (%) |
| --- | --- | --- | --- | --- | --- | --- | --- |
| 1. Title | 0.5 | 0.5 | 1 | 1 | 0.5 | 1 | 75.0% |
| 2. Abstract | 0.5 | 0.5 | 0.5 | 1 | 1 | 1 | 75.0% |
| 3. Background and objectives | 1 | 0.5 | 0.5 | 1 | 0.5 | 0.5 | 66.7% |
| 4. Health economic analysis plan | 0.5 | 0 | 1 | 1 | 0 | 1 | 58.3% |
| 5. Study population | 1 | 0.5 | 1 | 1 | 0.5 | 1 | 83.3% |
| 6. Setting and location | 1 | 1 | 1 | 1 | 1 | 1 | 100.0% |
| 7. Comparators | 1 | N.A.^a^ | 1 | 1 | N.A. | 1 | 100.0% |
| 8. Perspective | 0.5 | 0.5 | 0.5 | 1 | 0.5 | 1 | 66.7% |
| 9. Time horizon | 0.5 | 0 | 0.5 | 1 | 0 | 1 | 50.0% |
| 10. Discount rate | 0 | 0 | 0 | 1 | 0 | 0 | 16.7% |
| 11. Selection of outcomes | 1 | 1 | 1 | 1 | 1 | 1 | 100.0% |
| 12. Measurement of outcomes | 0.5 | 1 | 1 | 1 | 0.5 | 1 | 83.3% |
| 13. Valuation of outcomes | 0.5 | 1 | 1 | 1 | 0.5 | 1 | 83.3% |
| 14. Measurement and valuation of resources and costs | 0.5 | 1 | 1 | 1 | 0.5 | 1 | 83.3% |
| 15. Currency, price date, and conversion | 0 | 0 | 0 | 1 | 1 | 0 | 33.3% |
| 16. Rationale and description of model | N.A. | N.A. | N.A. | 1 | N.A. | N.A. | 100.0% |
| 17. Analytics and assumptions of model | N.A. | N.A. | N.A. | 1 | N.A. | N.A. | 100.0% |
| 18. Characterizing heterogeneity | 0 | 0 | 0 | 1 | 1 | 1 | 50.0% |
| 19. Characterizing distributional effects | 0 | 0 | 0 | 0 | 0 | 1 | 16.7% |
| 20. Characterizing uncertainty | 0 | 0 | 1 | 1 | 0 | 1 | 50.0% |
| 21. Approach to engagement with patients and others affected by the study | 0 | 0 | 0 | 0 | 0 | 0 | 0.0% |
| 22. Study parameters | 0.5 | 1 | 1 | 1 | 1 | 1 | 91.7% |
| 23. Summary of main results | 1 | 1 | 1 | 1 | 1 | 1 | 100.0% |
| 24. Effect of uncertainty | 0 | 0 | 1 | 1 | 0 | 1 | 50.0% |
| 25. Effect of engagement with patients and others affected by the study | 0 | 0 | 0 | 0 | 0 | 0 | 0.0% |
| 26. Study findings, limitations, generalizability, and current knowledge | 1 | 1 | 1 | 1 | 1 | 1 | 100.0% |
| 27. Source of funding | 1 | 1 | 1 | 1 | 1 | 1 | 100.0% |
| 28. Conflicts of interest | 1 | 1 | 1 | 1 | 1 | 1 | 100.0% |
| Total (%) | 51.9% | 50.0% | 69.2% | 89.3% | 54.0% | 82.7% |  |
| Quality categories^b^ | Insufficient | Insufficient | Very good | Excellent | Insufficient | Very good |  |

^a^N.A. = not applicable.

^b^Quality categories: Excellent scoring, ≥85.0%; very good scoring, 70.0%–84.0%; good scoring, 55.0%–69.0%; insufficient scoring, <55.0%.

Husereau D, Drummond M, Augustovski F, et al (2022) Consolidated Health Economic Evaluation Reporting Standards 2022 (CHEERS 2022) Statement: Updated Reporting Guidance for Health Economic Evaluations. Value Health, 25(1): p. 3-9. https://doi.org/10.1016/j.jval.2021.11.1351.
